# A rare case of anal condyloma in AIDS patient

**DOI:** 10.11604/pamj.2020.37.46.25397

**Published:** 2020-09-10

**Authors:** Alok Kumar Diwedi, Kiran Khandare

**Affiliations:** 1 Department of Shalyatantra, Mahatma Gandhi Ayurved College Hospital and Research Centre, Salod (H), Datta Meghe Institute of Medical Sciences, Wardha, India

**Keywords:** Anal condyloma, human immunodeficiency virus, peri anal growth

## Image in medicine

*A 55-year-old female patient came to outpatient department with complain of bleeding per rectum, swelling and itching at perianal region. Patient was known case of HIV and taking treatment for same. The clinical examination revealed perianal growth 6 cm long axis. The per rectal examination reveals internal hemorrhoid at 3, 7 and 11 o’clock. The patient had CD4 count of 254 elements/mm^3^ along with, the patient had Hb 9.2 mg/dl total leucocyte count 1200cu/mm. On clinical examination, patient was diagnosed with anal condyloma*.

**Figure 1:**
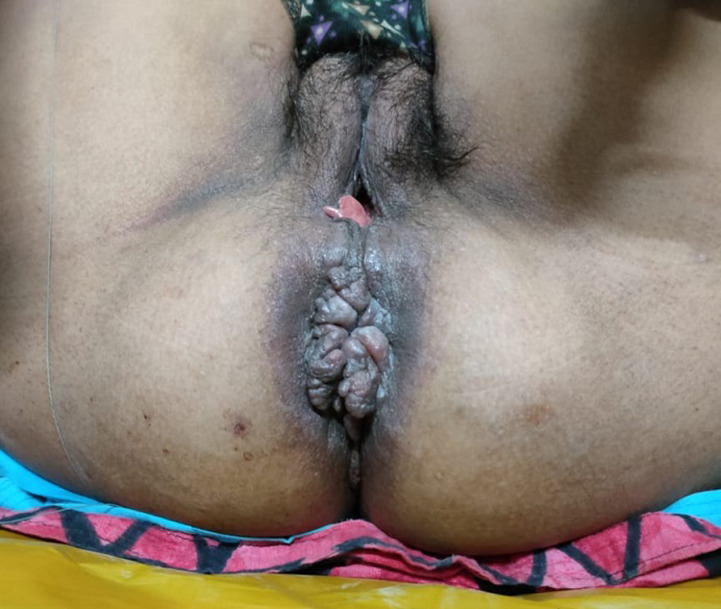
frontal view of anal codyloma in peri-anal region

